# An elderly low-grade fibromyxoid sarcoma patient with early postoperative recurrences and metastases: a case report and literature review

**DOI:** 10.3389/fmed.2024.1172746

**Published:** 2024-02-01

**Authors:** Xiaoyue Zhang, Yongkang Qiu, Jixin Zhang, Zhao Chen, Qi Yang, Wenpeng Huang, Lele Song, Lei Kang

**Affiliations:** ^1^Department of Nuclear Medicine, Peking University First Hospital, Beijing, China; ^2^Department of Pathology, Peking University First Hospital, Beijing, China

**Keywords:** low-grade fibromyxoid sarcoma, postoperative recurrences, metastasis, magnetic resonance imaging, PET/CT

## Abstract

**Background:**

Low-grade fibromyxoid sarcoma (LGFMS) is a rare type of soft tissue sarcoma that often involves the deep soft tissue of the extremities and trunk in young and middle-aged adults. It is uncommon in the elderly. Here we discuss a case of LGFMS in an elderly patient who had recurrence and metastasis within 2 years of resection of the primary tumor.

**Case report:**

A 71-year-old LGFMS patient was presented with a mass in the left forearm accompanied by pain and numbness from the left upper arm to fingers. The patient subsequently underwent 3 surgical resections, although she had 3 recurrences within 6 months after the initial diagnosis. Considering the malignant biological behavior of the tumor, an amputation at 5 cm above the elbow was eventually performed. However, recurrence in the extremity of the stump and chest wall metastasis were observed 2 years after amputation. Then resection of the metastases, radiotherapy and particle implantation therapy were performed. The patient is currently undergoing follow-up and has no evidence of recurrence.

**Conclusion:**

In our case, multiple early postoperative recurrences may be associated with a positive margin at initial operation. The patient underwent a total of 5 operations including local resection of the primary tumor, twice wide resections, amputation and metastatic surgery with 4 early postoperative recurrences and metastases within 4 years, suggesting that LGFMS may have highly invasive biological behavior. Our case demonstrated that early aggressive surgical treatment is recommended for LGFMS patients with a positive margin at initial operation and patients who had recurrence even after wide resection rather than local resection. Further research is needed to develop more effective treatment options for rapidly progress and highly aggressive LGFMS.

## Introduction

Low-grade fibromyxoid sarcoma (LGFMS) is a rare type of soft tissue sarcoma that is classified as a “fibroblastic/myofibroblastic tumor” according to the 2020 edition of the World Health Organization (WHO) Classification of Bone and Soft Tissue Tumors. LGFMS often involves the deep soft tissue of the extremities and trunk in young and middle-aged adults with a deceptively benign histologic appearance (1). Its biological behavior is both indolent and malignant. About 9% of patients have recurrence and 6% have metastasis within 2 years after resection of primary tumor (2). However, the long-term local recurrence rate and the eventual metastasis rate can reach 64 and 46%, and the clinical course is often prolonged (3). Here, we described a rare case of a 71-year-old LGFMS patient who underwent a total of 5 operations with 4 early postoperative recurrences and metastases within 4 years. In addition, we summarized characteristics of LGFMS cases with twice or more local recurrences by reviewing previous relevant literature in order to assist the clinician in decision making and treatment planning for achieving a good prognosis for this rare tumor.

## Case presentation

**Figure 1:**
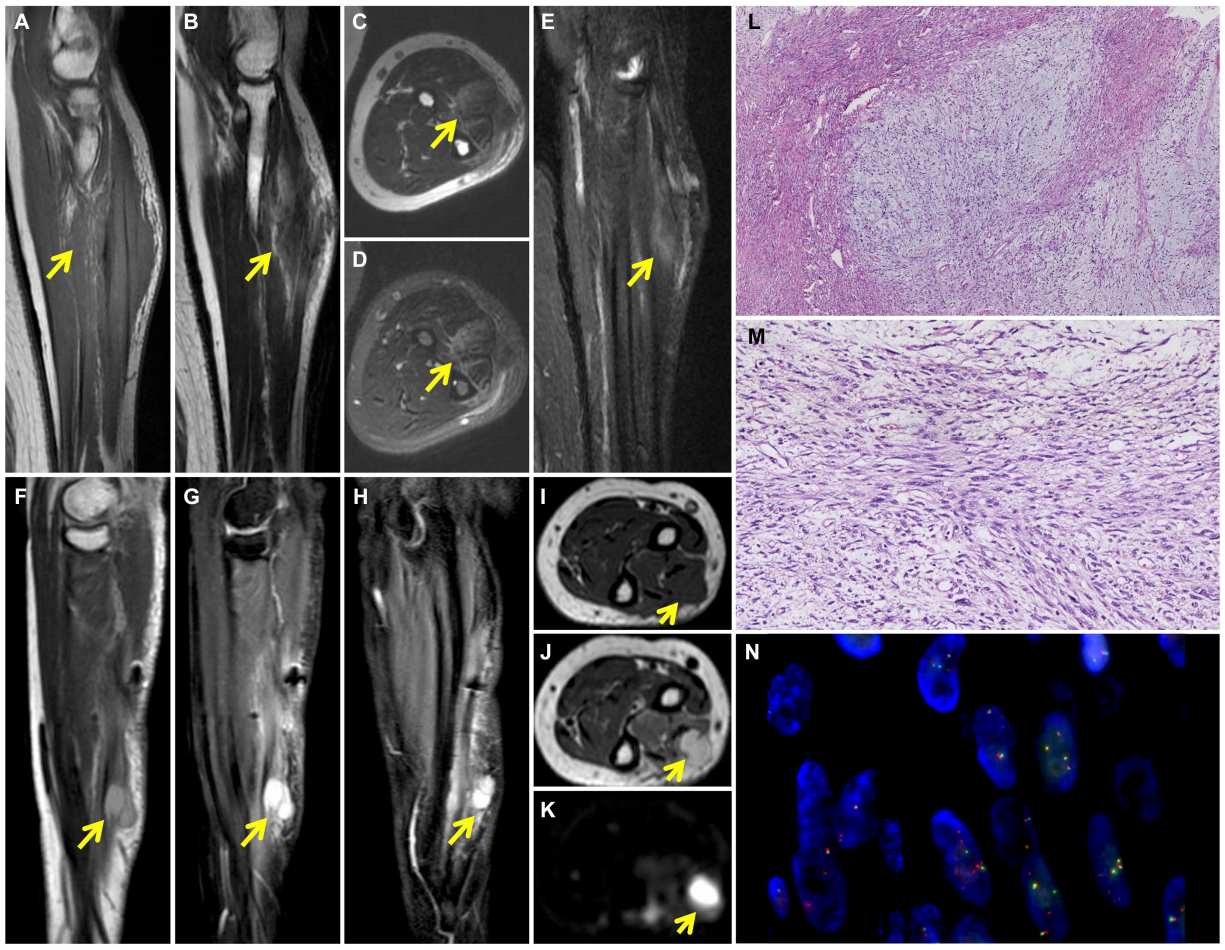
Preoperative MRI shows the soft tissue mass in the left arm. T1-weighted coronal image **(A)** shows patchy and irregular isointense signal lesion (yellow arrow). The soft tissue mass (yellow arrow) shows slightly high signal intensity on T2-weighted coronal **(B)** and axial **(C)** images and high signal intensity on T2 fat-suppressed axial **(D)** and coronal **(E)** images. 6-months postoperative MRI shows recurrence of left arm LGFMS after initial resection. Multiple nodules (yellow arrow) were detected in the extensor digitorium at the medial of the ulna, which exhibited high signal intensity on T2-weighted sagittal **(F)**, T2 fat-suppressed sagittal **(G)** and coronal **(H)** images. The nodules (yellow arrow) demonstrate low signal intensity on T1-weighted axial image **(I)**, high signal intensity on T2-weighted **(J)** and diffused **(K)** weighted axial images. Photomicrograph shows the alternating myxoid and fibrous area in the lesion **(L)** (HE, original magnification × 40). Higher magnification shows the spindle-shaped tumor cells arranged in bundles with uneven proliferation **(M)** (HE, original magnification × 200). FISH analysis of *FUS* detected both fused-and split-signals **(N)**.

A 71-year-old female patient presented with a mass in the left forearm for 3 years. When the patient sought medical attention 1 year ago, the doctor considered a diagnosis of fibroma and did not proceed with surgical treatment. The mass began to enlarge and harden significantly more than 5 months ago, accompanied by pain and numbness from the left upper arm to fingers. The patient had no family history of a similar condition. Physical examination revealed an 8 cm × 6 cm tender fixed mass on the patient’s left forearm with an unclear boundary. Laboratory tests showed no obvious abnormalities. Magnetic resonance imaging (MRI) was performed to further assess the lesion. The pronator teres, flexor carpi radialis, flexor digitorum superficialis, flexor carpi ulnaris and intermuscular space showed patchy and irregular isointense signals on T1-weighted image (Figure 1A) and high signal intensity on T2-weighted image (Figures 1B–E). The boundary of the lesion is not clear, with intermuscular edema (Figures 1A–E).

The patient subsequently underwent a resection of the left forearm mass with a size of 8 cm × 6 cm × 6 cm. Intraoperative findings showed the mass borders were indistinct from surrounding muscle fibers and tendon. The trunk and branches of the dorsal interosseous nerve were also tightly adhered to the tumor and the nerve branches surrounded by tumor tissue were removed. Postoperative histopathology showed alternating myxoid and fibrous areas and that the tumor cells were predominantly spindle-shaped cells arranged in bundles with uneven proliferation. Moderate cytologic atypia and up to 11 mitoses per 10 hpf in the most active part of the tumor were observed (Figures 1L,M). By immunohistochemistry (IHC), the tumor cells were diffusely positive for vimentin, focally for SMA, KP1, MSA, and negative for S^−100^, CD34, EMA, HMB-45, desmin, LCA, AE1/AE3, ALK (1A4), SOX10, TLE1 and ß-catenin. Ki-67 staining showed the proportion of the positive tumor cells was about 20%. Upon pathologic examination with immunohistochemistry, low-grade soft tissue sarcoma was diagnosed. Using the French Federation of Cancer Centers Sarcoma Group (FNCLCC) guidelines for the histopathologic grading, the final scores were 4 and the tumor was graded as 2. The tumor cells infiltrated surrounding skeletal muscle tissue. Presence of tumor cells at the surgical resection margin can also be observed. Genetic test was performed to differentiate between low-grade fibromyxoid sarcoma and low-grade/well-differentiated myxofibrosarcoma. *In situ* hybridization (ISH) and fluorescence *in situ* hybridization (FISH) detected *FUS* gene rearrangement, which was consistent with the diagnosis of LGFMS (Figure 1N).

**Figure 2:**
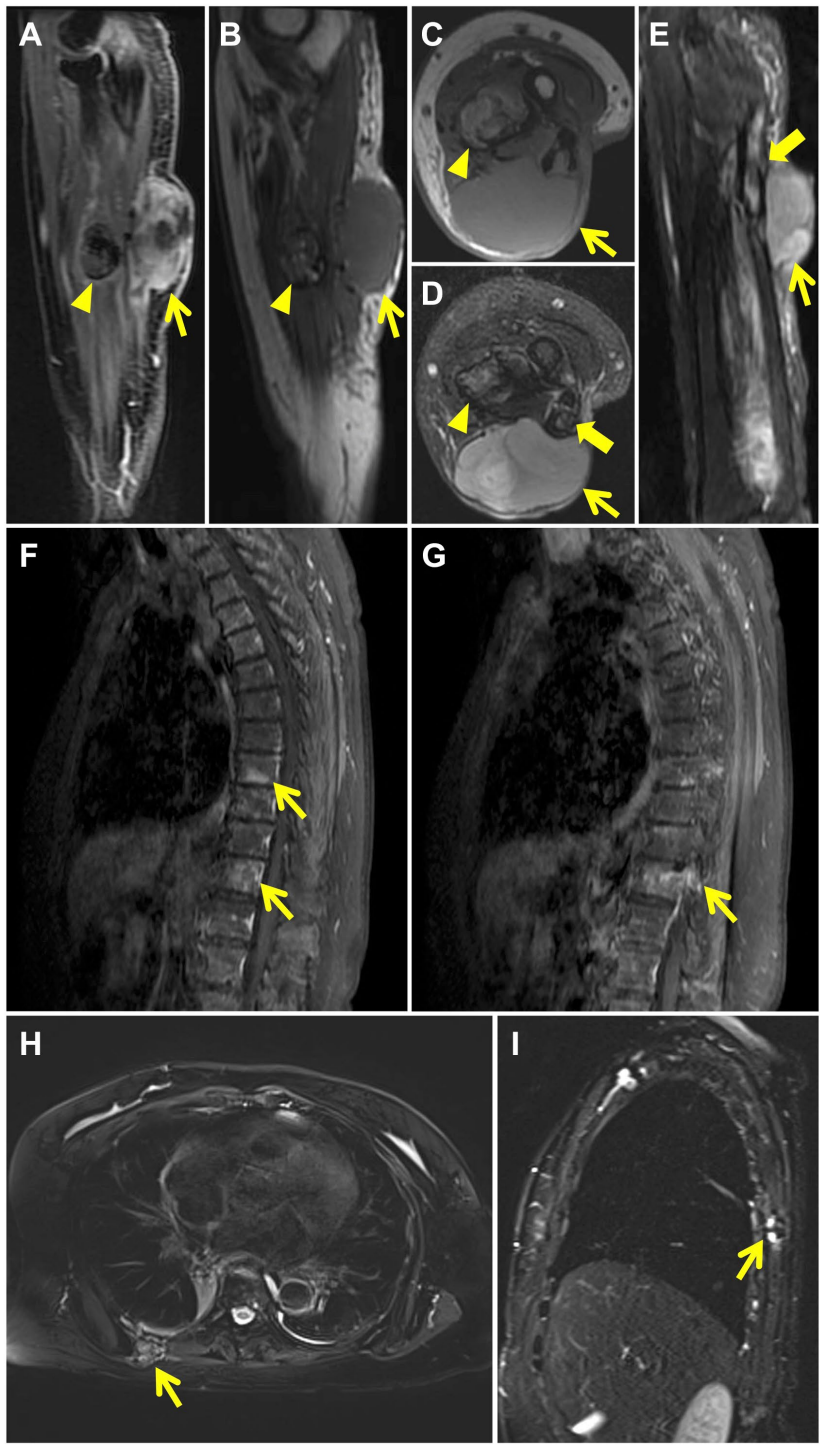
MRI after wide resections shows recurrence of left arm LGFMS. The mass in the extensor carpi ulnaris (thin yellow arrow) demonstrates high signal intensity on contrast enhanced T1 weighted sagittal image **(A)** and isointense signal intensity on T1-weighted sagittal **(B)** and axial **(C)** images. T2 fat-suppressed axial **(D)** and sagittal **(E)** images reveal high signal intensity in the mass (thin yellow arrow) and in the cavity of the left ulna with discontinuous cortical bone (thick yellow arrow). The nodule in the flexor digitorum profundus (yellow arrow head) demonstrates rim enhancement on contrast enhanced T1 weighted sagittal image **(A)** and mixed signal intensity on T1-weighted sagittal **(B)** and axial **(C)** images. T2 fat-suppressed axial image **(D)** reveals mixed high signal in the nodule (yellow arrow head). Images of contrast-enhanced MRI of the thoracic spine after wide resections show patchy high signal intensity in the T8, T11 vertebral body **(F)** (thin yellow arrows) and abnormal enhancement in T11 vertebral body and accessory **(G)** (thin yellow arrow). MRI after amputation shows chest wall metastases. T2 fat-suppressed axial **(H)** and sagittal **(I)** images reveal a mass with mixed signal intensity in the right back muscle (thin yellow arrow) and show discontinuous bone cortex of the adjacent right 7th rib with high signal intensity (thin yellow arrow).

**Figure 3:**
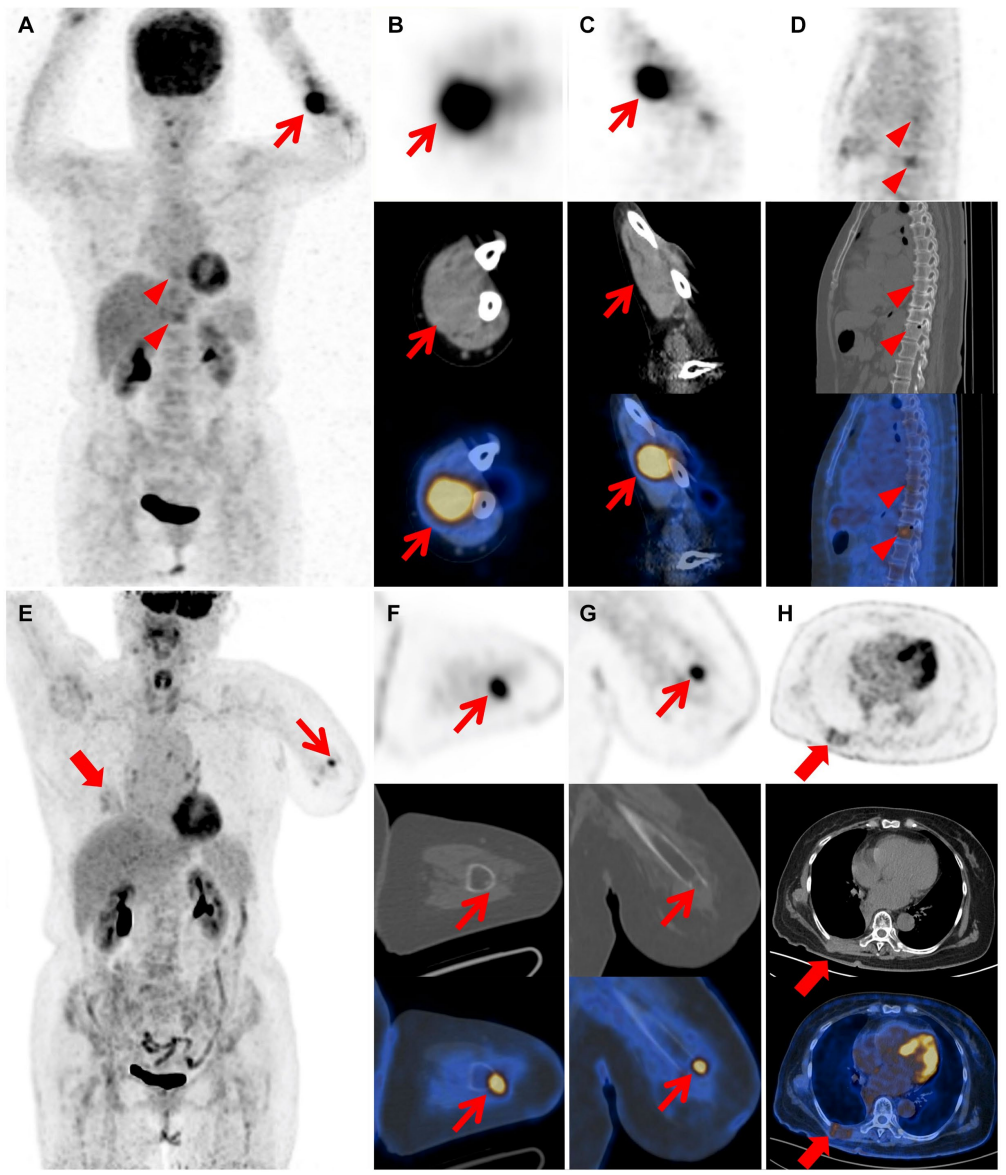
Images of ^18^F-FDG PET/CT after wide resections and PET/CT after amputation. The MIP image **(A)** shows left forearm (red arrow) and T8, T11 vertebral body lesions (red arrow heads) with varying degrees of FDG uptake. The coronal **(B)** and axial **(C)** views of mass in the left forearm demonstrate increased metabolic uptake (red arrow; SUVmax 11.2). The sagittal image **(D)** demonstrates increased metabolic uptake in the T8 and T11 vertebral body (red arrow heads; SUVmax 3.3, 4.1), suggesting the presence of bone metastasis. The MIP image **(E)** reveals absence of the left forearm and lesions of the left stump (thin red arrow) and chest wall (thick red arrow) with varying degrees of FDG uptake. The axial **(F)** and coronal **(G)** views of the left stump demonstrate increased metabolic uptake at the extremity (thin red arrow; SUVmax 5.4). The axial image **(H)** reveals irregular thickening of the right chest wall with mildly increased metabolic uptake (thick red arrow; SUVmax 2.9).

Two months after the primary surgery, MRI revealed multiple subcutaneous and intermuscular soft tissue nodules in the left forearm (Figures 1F–K). The patient subsequently had a wide resection of the local recurrent tumor with a 2 cm margin in all directions to the mass or the scar from the previous surgery. The postoperative histopathological findings reveal a proliferation of spindle-shaped tumor cells, with some arranged in a fascicular pattern. These cells exhibit moderate to severe cytologic atypia, and up to 31 mitoses per 10 hpf in the most active regions were observed. A mucinous background is evident in certain areas of the stroma. Three months after the second surgery, the patient found a mass in the left forearm again. MRI also showed multiple intermuscular and subcutaneous soft tissue nodules and abnormal signals at the left ulna and proximal radius. Then, the patient underwent another larger-scale resection. The postoperative histopathological features exhibit resemblances to those observed in the preceding recurrence. After the wide resections, the follow-up MRI showed multiple masses in the left forearm muscle (Figures 2A–E) and abnormal signals in the T8, T11 vertebral body (Figure 2F) and accessory of T11 (Figure 2G). To assess the patient’s whole-body situation, ^18^F-flurodeoxyglucose (^18^F-FDG) positron emission tomography/computed tomography (PET/CT) examination was further performed. It revealed increased metabolic uptake with a maximum standardized uptake value (SUVmax) of 11.2 within the left forearm mass (Figures 3A–C) and increased FDG uptake in the T8 and T11 vertebral body (SUVmax 3.3, 4.1) where slightly lower bone mineral density was observed (Figures 3A,D). Then, her left arm was amputated 5 cm above the elbow and the mass was about 10 cm from the incision, protruding from the skin with a necrosis surface. The histopathological characteristics of the tumor manifest similarities to those observed in prior recurrences. IHC assessment demonstrated diffusely positive for vimentin, focally for CD34, CD31, and negative for S^−100^, SMA, desmin, AE1/AE3. Ki-67 staining showed the proportion of the positive tumor cells was 90%.

Two years after amputation, the patient was found to have a mass on the back. MRI showed a mass in the right back muscle (Figure 2H) and the adjacent right 7th rib (Figure 2I). PET/CT revealed increased FDG uptake at the extremity of the left stump (SUVmax 5.4; Figures 3E–G), irregular thickening of the right chest wall and bone destruction of the adjacent right 8th rib (Figure 3H). Resection of the metastases in the right chest wall and the 8th rib was performed on the patient. She was also treated with radiotherapy and particle therapy. The patient is currently undergoing follow-up and has no PET/CT evidence of recurrence.

## Discussion

LGFMS was first reported by Evans (1) in 1987, which has a deceptively benign histologic appearance and relatively frequent recurrence and metastasis. In Evans’ early reports, 7 out of 12 LGFMS patients developed distant metastasis, with a follow-up ranging from 5.5 to 50 years (4). However, among the 54 LGFMS patients reported by Folpe et al. (2), only 5 (9%) had local recurrence and 3 (6%) had distant metastasis, possibly due to the fact that most of the patients had been treated with aggressive surgery or the relatively short follow-up time, with a median of 24 months. In 33 cases of LGFMS later reported by Evans, the interval to local recurrence was up to 15 years with a median of 3.5 years, while the interval to metastasis was up to 45 years with a median of 5 years. (3) These reports suggest that LGFMS is characterized by late recurrence and metastasis with a prolonged clinical course, therefore a short-term follow-up may not be sufficient to assess the biological behavior of this tumor. In our case, pathology indicated a positive surgery margin upon the resection of primary mass. Local recurrence occurred 2 months later and a wide resection was performed. However, local recurrence and bone metastasis were found 1 month later. The patient underwent radiotherapy, particle therapy and amputation. She was regularly followed up for 2 years without recurrence. Two years after the amputation, PET/CT scans revealed increased FDG uptake at the extremity of the stump and metastatic lesions in the back that invaded adjacent ribs. The patient had to undergo another resection of the metastatic lesion in the chest wall. This patient had 3 recurrences within 6 months of the initial diagnosis, underwent a total of 5 surgeries within 4 years, which is probably the most detailed report of the fastest recurrence with the shortest interval of LGFMS so far.

We identified 19 LGFMS cases with twice or more local recurrences based on the literature search on PubMed from 1987 to 2022. The keywords were “low grade fibromyxoid sarcoma.” The characteristics of all cases are summarized in Table 1. The median age of the patients was 26 years (range, 6–53 years). Tumor size (maximum diameter) was known in 10 cases, in which it ranged from 3.5 to 15 cm with a median of 10 cm. Among the 19 patients, 13 were men, with a male-to-female ratio of 2.17:1. All patients received surgery as initial treatment, only 3 had data on surgical margin status, all of which were positive. The median time of first recurrence was 3 years while the median time from illness onset to death was 28 years. Five patients had metastases, including lung (*n* = 5), bone (*n* = 1) and chest wall (*n* = 1). Characteristics of dedifferentiated sarcoma were observed in 2 recurrent tumors and features of sclerosing epithelioid fibrosarcoma (SEF) was observed in one case.

**Table 1:** Characteristics of LGFMS cases with twice or more local recurrences.

Author	Case no.	Age/Sex	Site	Maximum primary tumor site(cm)	Initial treatment	Follow up
Goodlad et al. (5)	1	53/M	Trunk (anterior chest wall)	NA	Excision (margin unknown)	LR at 4, 6, 7, 9 and 10 years
	2	50/M	Groin	5	Excision (margin unknown)	LR at 2, 9 and 10 years
	3	18/M	Lower limb (thigh)	NA	Excision (margin unknown)	LR at 7, 11 and 15 years
	4	53/M	Trunk (anterior chest wall)	13	Excision (margin unknown)	LR at 2 and 5 years
Oda et al. (6)	1	13/M	Buttock	NA	Excision (margin unknown)	LR at 2, 3, 5 years; wide excision; NED at 63 months
Evans et al. (3)	1	28/F	Axilla-chest wall area	11	Excision (margin unknown)	Lung metastases at presentation. LR at 3, 26, 30 years, showed features of SEF. Alive at 31 years.
	2	6/F	Inguinal area	3.5	Excision (margin probably +)	LR at 13, 19, 25, 30 and 39 years. Lung metastases at 45 years. Alive at 70 years.
	3	30/M	Perineum	>6	Excision (margin unknown)	Recurrences excised 17 times over a 29-year period. Died of recurrent at 31 years.
	4	51/M	Inguinal area	13.5	Excision (margin unknown)	LR at 5, 9 years. Lung and bone metastases. Died postoperatively at 9 years.
	5	26/M	Small bowel mesentery	15	Excision (margin +)	LR at 3 and at 10 years. Died of recurrence at 28 years.
	6	26/M	Neck	NA	Excision (margin unknown)	LR at 3, 10, 14, 22, 24, 27, 31, 36 and 43 years. Alive and NED at 44 years.
	7	38/M	Buttock	NA	Excision (margin unknown)	LR at 4, 6, 12, 14, 21, 23 and 28 years. Biopsy showed dedifferentiated sarcoma. Died at 31 years.
	8	22/F	Abdominal wall (lower)	NA	Excision (margin unknown)	LR at 3, 6 and 11 years. Lung metastases. Alive at 12 years.
	9	9/M	Neck	NA	Excision (margin unknown)	LR at 5, 9, 12, 17, 20, 22 and 23 years. Died of lung and chest wall metastases at 42 years
	10	24/F	Inguinal area	NA	Excision (margin unknown)	LR at 4 and at 14 years. Alive and NED at 19 years.
	11	39/M	Mesentery	10	Excision (margin unknown)	LR at 2 and at 18 years. Alive at 18 years.
	12	41/F	Chest wall	10	Excision (margin probably +)	LR at 1, 2 and 4 years. Died at 6 years.
	13	26/M	Retroperitoneum	10	Excision (margin unknown)	Recurrent dedifferentiated tumor excised at 2 years and more recurrent tumor shortly after. Died at 6 years.
Indap et al. (7)	1	20/F	Shoulder	NA	Excision (margin unknown)	LR at 3 and 20 years. Alive at 22 years.

LGFMS tends to occur in young and middle-aged people. It often involves the deep soft tissues of lower limbs, followed by the chest wall, shoulder, groin and other areas. The most common site of metastasis is the lung. It is histologically characterized by alternating myxoid and fibrous areas, bland fusiform cells, a whorled growth pattern (6). Other histological variations include giant rosettes (8), areas of hypercellularity, foci with rounded epithelioid cells, less commonly observed are focal osseous metaplasia and significant nuclear pleomorphism. In addition, characteristics of focal sclerosing epithelioid fibrosarcoma has been reported in LGFMS (9). However, the relationship between these histological variations and tumor biological behavior has not been reported. In our case, focal dense cells with less stroma and higher atypia were found in the resected recurrent tumor, which may be related to its rapidly proliferation and aggressive behavior.

Most LGFMS appear to be well-circumscribed but have no capsule. Local infiltration may occur, and resection is usually incomplete (10). Therefore, the preferred treatment for LGFMS is wide resection and radical surgery (11, 12). Prognosis of LGFMS is based on tumor size at diagnosis, invasion to adjacent tissues, and surgical margin (3, 4). Patients with negative margins after active surgical resection have a lower recurrence probability and a longer recurrence interval (13). Our case had a positive surgical margin, which may account for the multiple early postoperative recurrences. If possible, wide resection should be the preferred treatment for all local recurrences (14). There is currently little guidance on how to treat LGFMS patients with metastatic disease. Due to the very low mitotic rate in LGFMS, chemotherapy and radiotherapy usually have no significant effect on the long-term prognosis (12). When bone metastases occur, especially vertebral metastases, local radiotherapy and particle therapy may be alternative treatments to reduce skeletal related events, while radical surgery for distant metastases (usually to the lungs) may still be the best option for patients. The efficacy of conventional systemic therapy for advanced LGFMS is also limited (15). A recent study suggested that Trabectedin could be effective on metastatic patients (16), which may be related to the *FUS::CREB3L2* fusion gene in LGFMS.

In our case, the tumor progressed rapidly with early postoperative recurrences and metastases. These clinical manifestations along with histopathological findings of focal dense cells and less stroma are similar to SEF. It is necessary to distinguish LGFMS from SEF. SEF, originally reported by Meis-Kindblom et al. (17), is characterized by a large number of sclerotic stroma and rounded or polygonal epithelioid cells growing in cords or nests. In a study of LGFMS, Guillou et al. (18) included 4 cases of SEF as a comparison, one of which was similar to LGFMS. Despite the short follow-up of these SEF patients, 3 had metastases at presentation and 4 had local recurrence at 6 months. SEF often occurs in older people, metastasizes more frequently, and has a higher mortality rate and shorter overall survival than LGFMS. Antonescu et al. (19) mentioned that a few SEF tumors had fibroma-like areas and myxoid components similar to LGFMS. When the typical morphological features of SEF and LGFMS appear simultaneously or sequentially, it is called hybrid SEF/LGFMS (17, 20, 21). Compared with LGFMS, hybrid SEF/LGFMS exhibit more aggressive tumor behavior, and the recurrence, metastasis and death caused by tumors occur earlier and at shorter intervals. The histological morphology and clinical features of LGFMS and SEF partially overlap but also differ to some extent, which may be related to their common and unique genetic characteristics (16, 22–25). Based on these findings, we speculate that SEF may be a variant of LGFMS rather than a distinct fibrosarcoma under certain circumstances.

Pure SEF and hybrid SEF/LGFMS SEF are extremely rare, and therefore very limited data about the clinical behavior and the effectiveness of different treatments are known. Surgery remains the mainstay of treatment, especially wide resection with histologically negative margins. Perioperative or postoperative radiotherapy can be used due to the rapid growth and relatively more aggressive clinical features of pure SEF or mixed SEF/LGFMS, which may help to control tumor recurrence and metastasis, although its efficacy has not been proven by previous studies (26). A recent study suggests that chemotherapy has very limited efficacy in SEF (27).

^18^F-FDG PET/CT is a wildly used imaging modality in oncology. Metabolically active, high-grade soft tissue sarcomas tend to have high uptake of ^18^F-FDG on PET/CT. In our case, increased metabolic uptake was observed within the recurrent tumor (SUVmax 11.2), which could be related to aggressive tumor biology and frequent postoperative recurrences. Yoshimura et al. (28) reported a case of primary pulmonary LGFMS with ^18^F-FDG PET/CT findings suggestive of malignancy. Preoperative ^18^F-FDG PET/CT showed focal FDG uptake, with a maximum standardized uptake value of 5.59 in the mass. In addition, ^18^F-FDG PET/CT could also provide a whole-body assessment of the patient as part of tumor monitoring and follow up.

The recurrent tumors in the elderly patient we reported had histopathological findings of focal dense cells, less stroma, and high atypia. Thus, the possibility of mixed SEF/LGFMS being the diagnosis cannot be excluded. Our patient suffered multiple early postoperative recurrences and bone invasion, reflecting the biological behavior characteristics of rapid growth and strong invasion potential. Long-term follow-up should be performed for LGFMS patients with a positive margin at initial operation and patients who had recurrence even after wide resection to closely monitor recurrence or metastasis. The preferred treatment is early wide resection to reduce local recurrence. For metastatic lesions, radical excision should be performed if possible. Our case expands our understanding of the biological behavior of LGFMS and provides clinical experience in diagnosis and treatment.

## Conclusion

In our case, multiple early postoperative recurrences may be associated with a positive margin at initial operation. The patient underwent a total of 5 operations including local resection of the primary tumor, two wide resections, amputation and metastatic surgery with 4 early postoperative recurrences and metastases within 4 years, suggesting that LGFMS may have highly invasive biological behavior. Our case expands our understanding of the biological behavior of LGFMS and provides clinical experience in diagnosis and treatment. Further research is needed to develop more effective treatment options for rapidly progressing and highly aggressive LGFMS.

## Data availability statement

The original contributions presented in the study are included in the article/Supplementary material, further inquiries can be directed to the corresponding author.

## Ethics statement

Written informed consent was obtained from the individual(s) for the publication of any potentially identifiable images or data included in this article.

## Author contributions

XZ and YQ: acquisition and analysis of the work, draft the manuscript, imaging data collection, and analysis. ZC: manuscript editing. QY, WH, and LS: formal analysis and resources. LK and JZ: supervision and writing—review and editing. All authors contributed to the article and approved the submitted version.
