# Supplementary figures and images for: An elderly low-grade fibromyxoid sarcoma patient with early postoperative recurrences and metastases: a case report and literature review
Source: Front Med (Lausanne). 2024 Feb 1;11:1172746. doi: 10.3389/fmed.2024.1172746 (PMC10867330; doi:10.3389/fmed.2024.1172746)

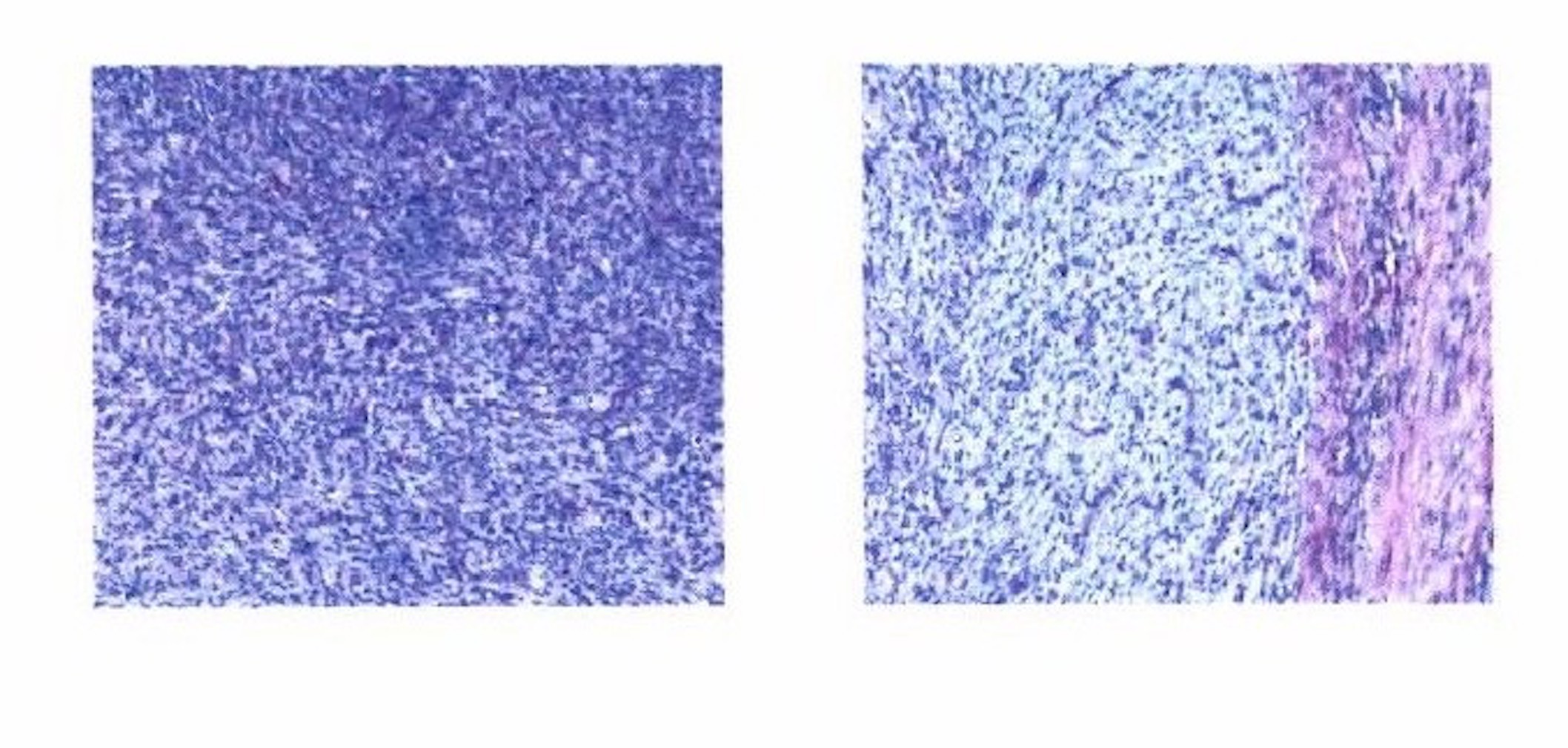

Supplement: Supplementary file 1 [file Image_1.JPEG]

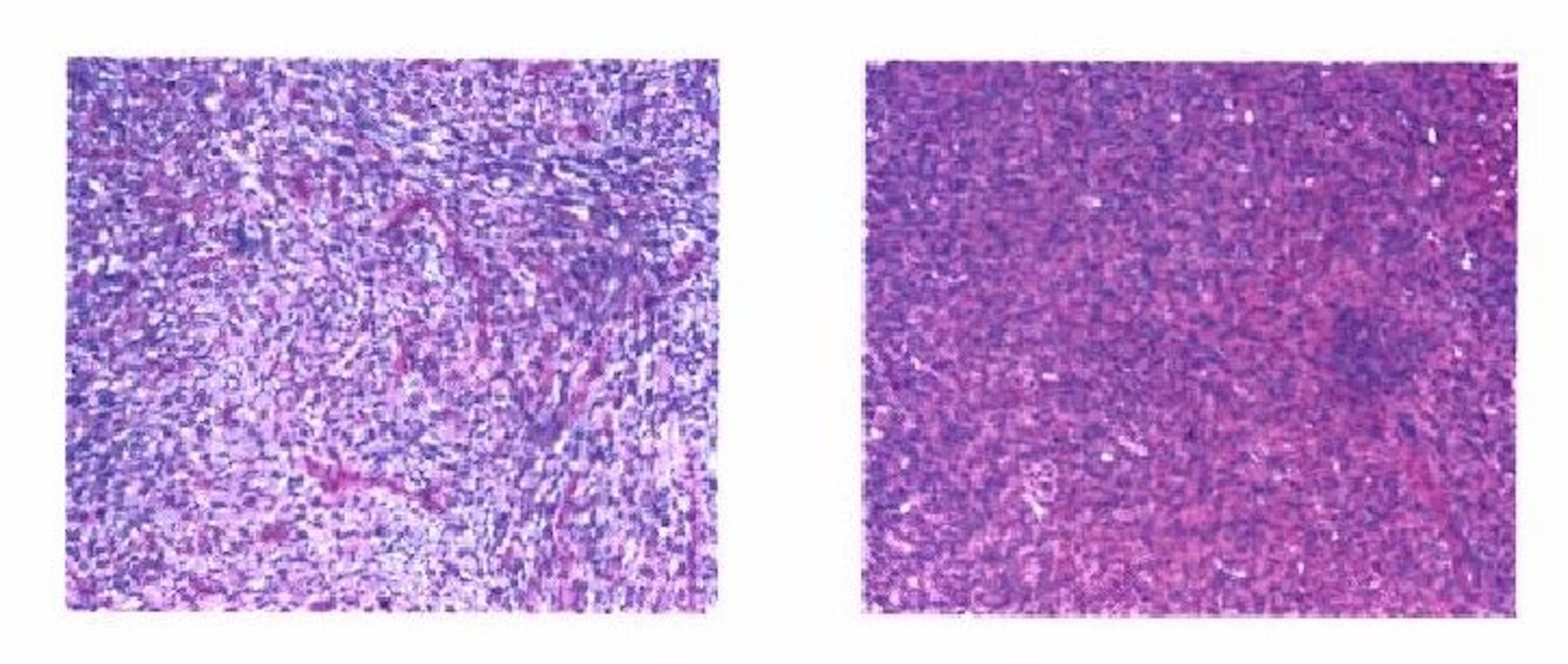

Supplement: Supplementary file 2 [file Image_2.JPEG]

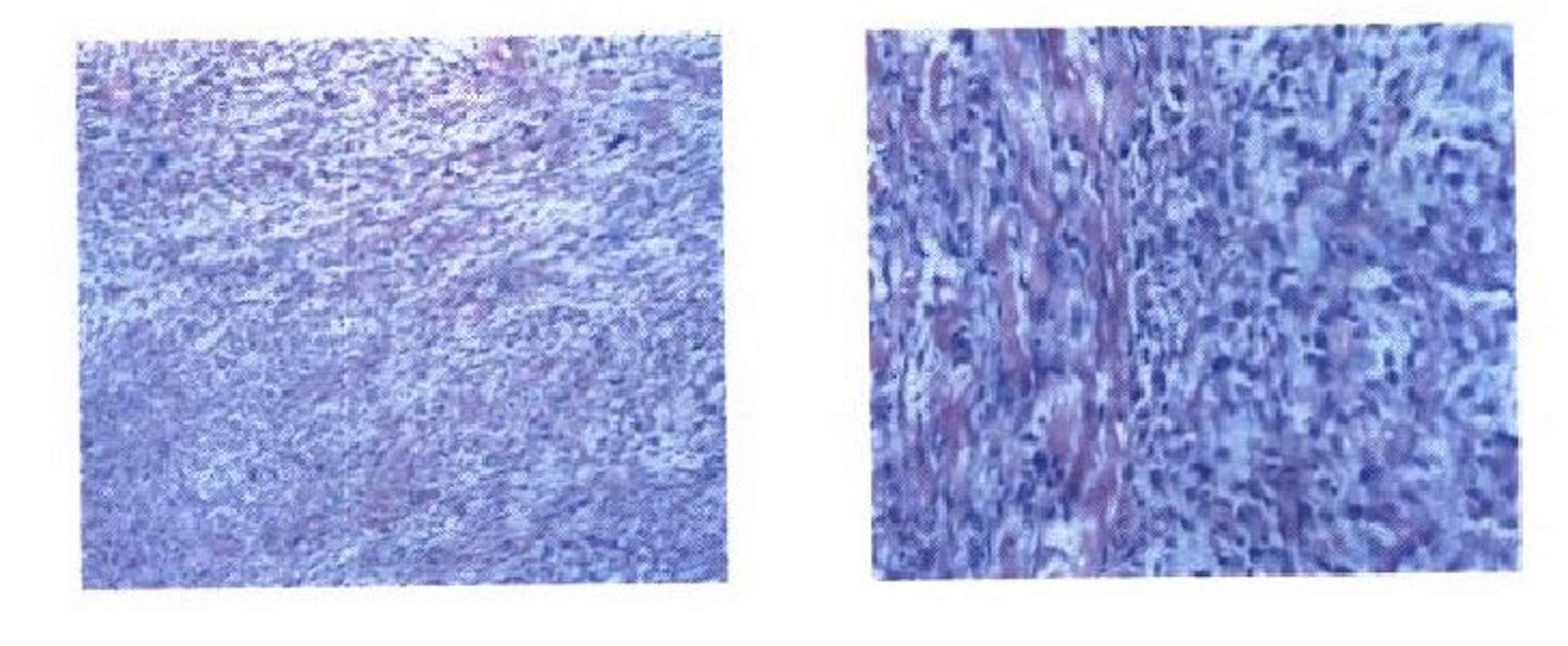

Supplement: Supplementary file 3 [file Image_3.JPEG]
